# Additive fabrication and experimental validation of a lightweight thermoelectric generator

**DOI:** 10.1038/s41598-023-37222-w

**Published:** 2023-06-20

**Authors:** Carlo Fanciulli, Hossein Abedi, Adelaide Nespoli, Roberto Dondè, Caterina La Terra, Francesca Migliorini, Francesca Passaretti, Silvana De Iuliis

**Affiliations:** 1 CNR – Institute of Condensed Matter Chemistry and Technologies for Energy, Via Previati 1/E, 23900 Lecco, Italy; 2 Department of Mechanical, Industrial, Manufacturing Engineering, University of Toledo, 2801 W. Bancroft St., Toledo, OH 43606 USA; 3 CNR – Institute of Condensed Matter Chemistry and Technologies for Energy, Via Cozzi 53, 20125 Milan, Italy; 4 Department of Energy, Politecnico di Milano, Via Lambruschini 4, 20156 Milan, Italy

**Keywords:** Energy science and technology, Engineering

## Abstract

We develop a thermoelectric generator based on catalytic combustion and operating in the low power range (up to 10 W). Considering the target of small-scale thermoelectric generators, the additive technique was chosen as an enabling technology to customize the different parts of the presented device. The generator consists of a hexagonal shaped combustion chamber coupled to commercial thermoelectric modules, water-cooled at the cold side. Thanks to the components design, heat transfer across each part of the system is properly driven enhancing the thermal management of the system. Moreover, in order to improve the overall efficiency, exhausts outlet is designed to promote heat recovery. The generator is characterized achieving an electrical power output close to 9 W in continuous regime, with an overall efficiency of 3.55%. The compact size, the light weight, the simple design and the reliability in continuous operating conditions are all promising features of the device described. Furthermore, the materials chosen for the device can suggest a way to fabricate cheaper heat exchangers, actually one of the main costs of the device development.

## Introduction

In the past centuries, the increasing development in industry, means of transport and more generally in the life quality has required a continuous and growing need for energy consumption. Nowadays, electricity can account for this request also thanks to the actual electric generation technology development. On the other hand, the electronic device technology with greater attention to their miniaturization is receiving higher interest in encouraging new technological innovations, such as the development of portable technology. This can represent a new challenge also for power generation technologies: a portable, fully reliable and long lasting electric power generation solution is an important issue to pursue. The main limitation of continuous and widespread use of mobile equipment is essentially related to electric batteries because of their limited working hours and consequently to the long time spent for the batteries to get recharged. Moreover, due to the limited number of recyclable components, in a future perspective of sustainability, the quantity of waste associated with batteries is going to become a critical problem to be managed. In addition, electronics are rapidly improving in terms of performance also thanks to the evolving design of the components. However, the increasing number of embedded functions and the required growing connectivity lead to an overall power need for these portable devices. As a consequence, these aspects are tracing the boundary conditions for the new challenge of portable power sources. Two are the main approaches to consider: the development of new, better performing batteries or the development of alternative solutions able to substitute, or at least support, actual battery solutions.

Due to its peculiar characteristics, thermoelectric technology is a suitable solution for the development of compact portable power generators. In fact, thermoelectric devices are based on solid state components having no moving parts, enabling a continuous electrical power production in the presence of a thermal gradient. As a consequence, they result to be suitable candidates as batteries back up or power sources for electronic equipment^1^. The electrical output of thermoelectric devices, being directly related to the applied heat flow, is a DC signal constant in time, which is a valuable feature for electronic devices^2^. A significant drawback of thermoelectrics is essentially related to its low conversion efficiency. This becomes a minor issue in the low power generation range, where no other efficient and reliable technologies are available^3^. Electronic devices offer an important opportunity for thermoelectric solutions due to low power and low voltage requirements. Moreover, the high reliability of thermoelectricity is a major characteristic that can make this technology one of the most competitive^4^.

For the development of a thermoelectric generator (TEG) as a portable solution, a small size and suitable power output are required. The small size can make the design of the system challenging, especially in terms of thermal management. As reported in the literature attention is paid to optimize the thermal circuit and in particular to match the thermal resistance of the heat exchangers with the one of the thermoelectric modules (TEMs)^5–7^. In^8–10^ it is described how minimizing the heat losses and optimizing the thermal design strongly affect the TEG system efficiency. Many works are reported in the literature on the development of different TEG configurations^8^. Typically, the TEM efficiency, directly related to the involved thermoelectric materials, scales down due to the internal thermal and electrical resistances of the device. In the same way, the thermal chain surrounding the TEM is responsible for a major drop down of the conversion efficiency^9^. As a result, the common thermoelectric generators developed with commercial TEMs based on chalcogenides, which limit the maximum operating temperatures, are capable of a conversion efficiency below 5%. This result can be obtained with an optimized design of the overall system and generally drops down when the system dimension scales down^10^.

The main goal of this work is the design of a compact TEG capable of an electrical power output matching the one of portable batteries to be used as a backup system for both direct power supply or on-demand charging system. The chosen heat source is based on the catalytic combustion of propane/air mixture, as also reported in other recent studies^11–14^. Catalytic combustion is of particular interest for the high power density of hydrocarbons and, at the same time, relatively low combustion temperatures^15^. Consequently, a more suitable thermal circuit of the TEG system is obtained with a lower thermal resistance at the heat source. In addition, low combustion temperatures allow a good coupling with commercially available TEMs, based on chalcogenide materials, which exhibit a significant limitation in high temperature operating conditions. The catalytic process also enables safe and stable combustion^14,15^ without hazardous free flame. Moreover, the large availability of liquid propane on the customer’s market (actually used for lighters) makes the use of this fuel particularly fruitful. This kind of solution has been recently explored by different research groups developing miniaturized TEGs^16^. Yoshida et al.^12^ design a small device based on catalytic combustion achieving high conversion efficiency using hydrogen as fuel. The system is based on a planar combustion chamber placed in between two non-commercial TEM directly assembled on the combustor external surfaces. Federici el al.^13^. also introduced the catalytic combustion of propane to scale down the size of the generator looking for a portable design: here the system, again design for a flat combustor, is coupled with a single TEM. Both the systems achieve a power output staying below 1 W, a critical limitation for a technological application. Another solution for compact TEGs based on catalytic combustion is proposed by Xiao et al.^14^, taking advantage of the safe and flameless combustion allowed by catalytic process for the integration of the TEG on a pipeline, managing the power supply to the electronic control units. Recently, Aravind et al.^17^ propose a generator based on non-catalytic propane combustor capable of one of the best electrical power densities reported for a compact device. Their architecture is close to the one already tested by Merotto et al.^15^ in a meso-scale device based on propane catalytic combustion. The challenge faced is to achieve an electrical power output able to be profitable as a supply system, downscaling the size of the overall device to make it available for integration.

In this work, a small-sized hexagon shaped TEG has been developed and produced through additive manufacturing (AM). This choice enables an optimization of the design, access to complex geometries in a compact configuration and the possibility to set specific features able to improve the thermal management of the device^18,19^. Due to its selective material consumption, AM method is a strong tool to effectively produce a TEG system with a final cost reduction, avoiding materials waste and improving production rates. In this context, this method results to be very useful also for the production of optimized heat exchangers. These are key elements in the development of an efficient TEG and have a significant impact on the system production costs^20^. Concerning the process advantages and the cost reduction perspective associated with AM technology^21^, the compact design proposed in this work opens an opportunity for device portability, actually critical for other devices proposed in the literature.

A further focus of this work is to achieve a high power density for the TEG. To date, there are many works focused on increasing of the electrical power production with respect to the total mass of the system for space^22^ or terrestrial applications^16^. The maximum power/mass ratio of 5.4 W/kg has been obtained for GPHS-RTG with 300 W power production and about 56 kg^22^. However, these systems are not designed to be portable.

The TEG presented here is characterized in terms of electrical power outputs. Efficiency is evaluated in all operating conditions to figure out the critical issues still to be faced. The results are presented and discussed with the aim to stress the key points of the developed device.

## Materials and methods

### The TEG system

**Figure 1:**
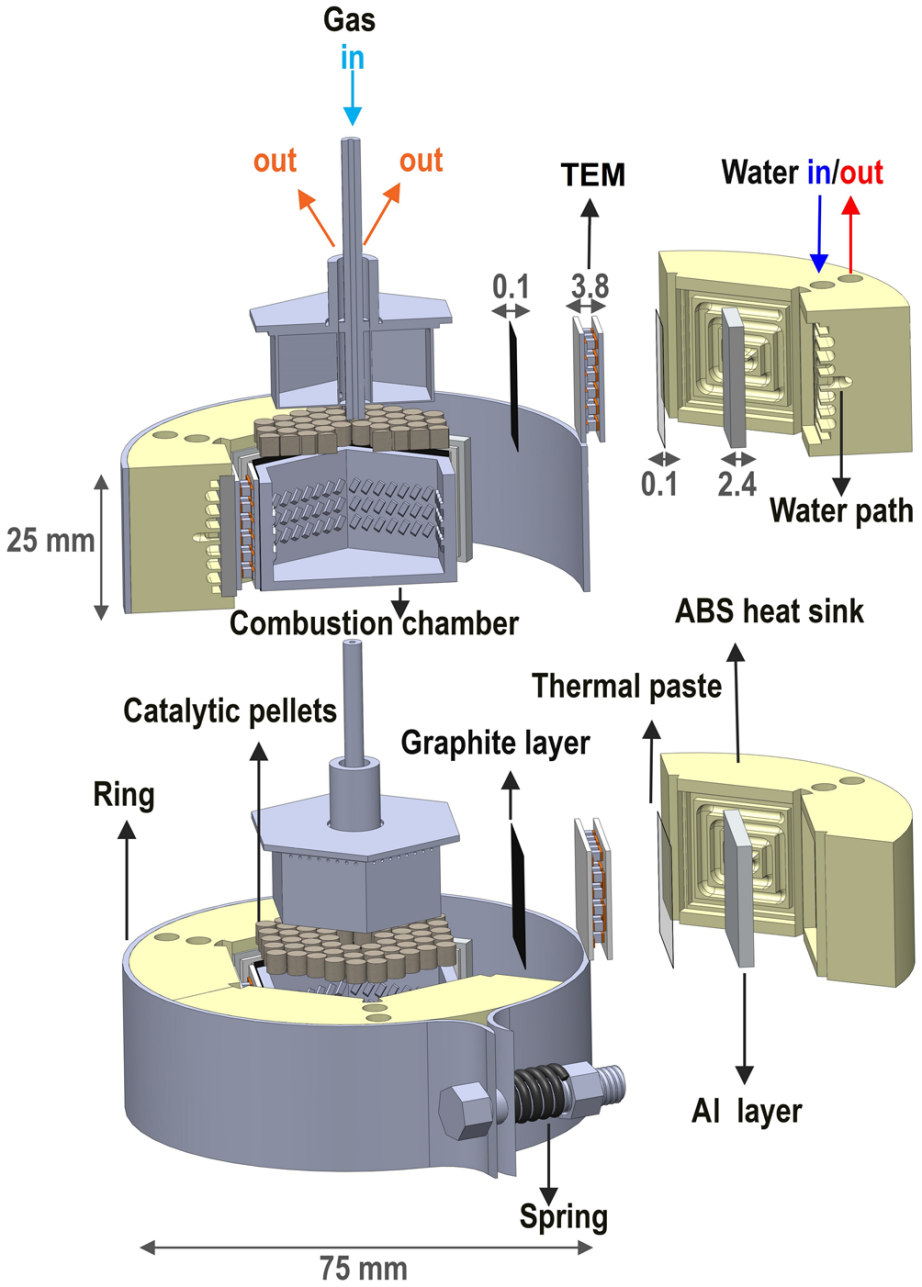
A schematic of the thermoelectric generator discussed in this work. Here, all the components of the thermal chain are shown.

Figure 1 reports the schematic of the TEG system. It consists of an hexagonal catalytic combustor used as the heat source, coupled with six 18 × 18 mm^2^ TEMs and six 18 × 18 mm^2^ water cooled cold plates. The hexagonal shape has been chosen because it allows the best coupling configuration between the heat source and the thermoelectric converting stage. Moreover, such a compact design allows the improvement of the output power density per unit volume. The design is properly chosen in order to achieve high fuel residence time in the chamber, wide gas exposure to the catalytic pellets, long time for heat exchange, resulting in an efficient heat transfer, suitable thermal management and improved combustion efficiency. The whole device is about 75 × 75 × 30 mm^3^ in size.

#### Thermoelectric modules

The TEMs used are commercial chalcogenide based devices (customized by European Thermodynamics)^23^. The modules are designed to continuously operate with the hot side temperature limited to 250 °C, suffering rapid materials degradation when a higher temperature is applied.

#### Combustor

The combustor is designed to promote the thermal exchange at the walls. It is composed of a chamber and a cap. The combustion chamber is filled with 52 pellets (Alumina cylinders with 1 wt.% of Pt on the surface, r = 1.6 mm, h = 3.2 mm) and fed with a propane/air mixture in stoichiometric ratio. The same pellets have been used in previous experiments^13^ obtaining optimal results both in terms of catalytic efficiency and lifetime. Fins on the inner walls of the chamber promote the thermal homogenization of the hot surface.

The cap is designed to drive the gases over the catalytic pellets positioned at the bottom of the combustion chamber and to conduct the hot gases through each of the six channels. Moreover, the peculiar construction allows the fresh gases in the inlet pipe to be heated up by the exhaust gases, improving the fuel ignition and combustion efficiency. The length of this stage has been adjusted in order to allow the preheating of the propane, effective to sustain a good rate for the catalysis, but preventing the undercooling of the exhausts. In fact, if exhausts temperature drops below 100 °C the water fraction condensate leading to a major issue related to burning instability.

**Figure 2:**
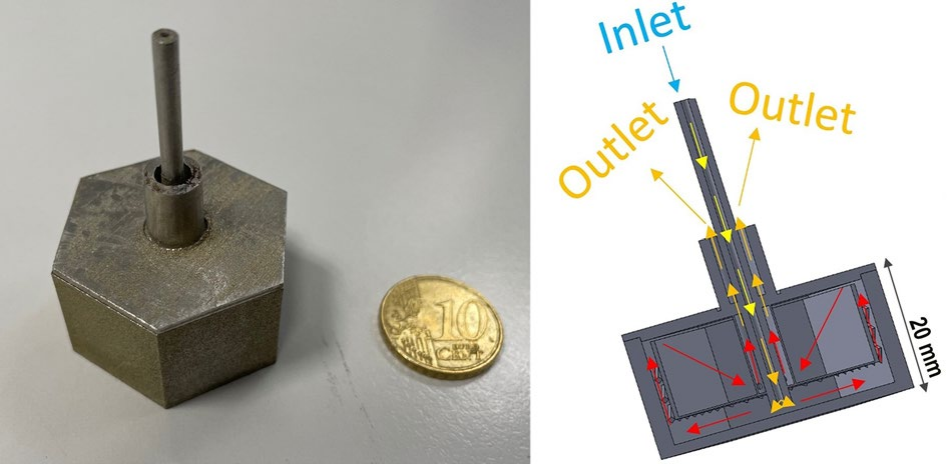
Photograph of the combustor and schematization of the gas path through the system.

The combustor is insulated by a refractory layer (ceramic on regular surfaces and wool on the others) to minimize the heat losses through convection and radiation. Figure 2 reports a photograph of the combustor and the gas path through the system. Photographs of the cap and of the assembled pellets into the combustor chamber are reported in the Figure S1.

The combustor chamber and the cap are produced with a laser powder bed fusion system (LPBF, AM400 of Renishaw), starting from gas-atomized micrometric powder (stainless steel 316L). The AM400 operates with a 400 W fiber laser in pulsed wave emission and a 70 µm spot diameter at the focal point. The AM400 selectively melts the powder according to a pre-defined CAD model, layer after layer. Before processing, a vacuum is pumped achieving an oxygen level lower than 500 ppm. The working chamber is then filled with Argon. No preheating is applied to the platform and parts are fabricated in a reduced build volume to optimize the powder consumption. Besides, the caps are properly oriented and supported to avoid the collapse of the structure during the process. The main LPBF process parameters are reported in Table 1.

**Table 1:** The main process parameters of the AM400 used to produce the combustor chamber parts.

Laser power	195 W
Layer thickness	50 μm
Hatch distance	110 μm
Point distance	60 μm
Exposure time	80 μs

Before the removal from the building platform, the components are thermally treated in the air at 450 °C for 2 h to release the residual stresses generated during the process. Subsequently, parts are removed from the building plate and post processed to refine the surfaces by performing sandblasting for the cells and mechanical polishing for the caps. Photographs of the combustion chamber and the cap on the building platform is reported in Figure S2.

#### Heat sink

**Figure 3:**
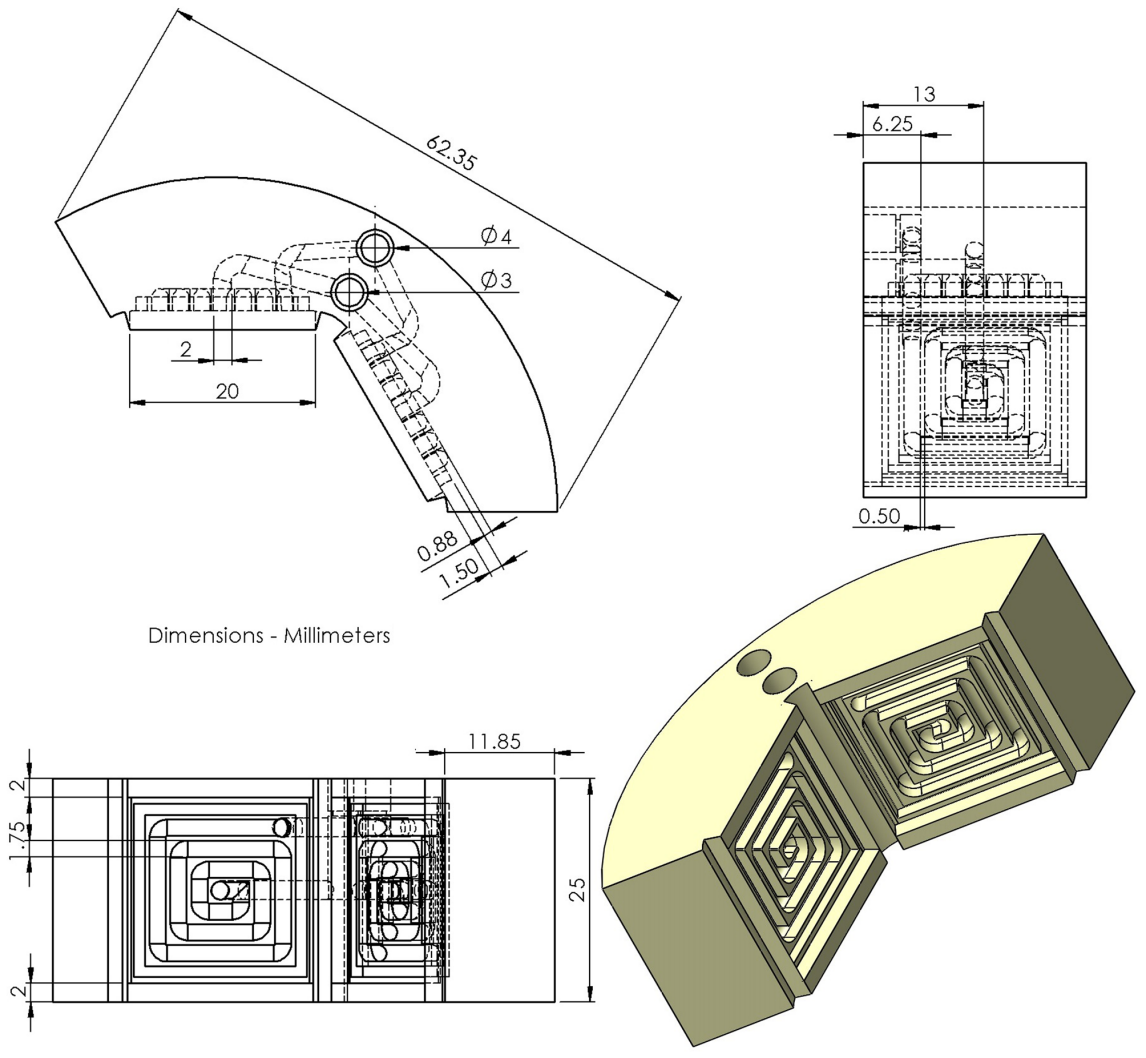
CAD design of the heat sink element. The component embeds the whole water circuit for the cooling of the TEMs surface and a small outside cave is used to seal the aluminum plate to the block.

The heat sink, shown as a sketch in Fig. 3, is 3D printed by Fused Deposition Modeling (FDM, Stratasys Dimension Elite model). The heat sink consists of three identical pieces building an external circular shape. This design eases the post processing stages, increases the system flexibility to be assembled and creates a round shape suitable for uniform pressure distribution over each TEM. The pieces are made of Acrylonitrile Butadiene Styrene (ABSplus) polymer. This material is chosen because of its stable mechanical properties even at temperatures close to 100 °C^24^. In fact, the design of the system requires the components to transfer the mechanical pressure to the thermal chain of the generator. The advantage of using additive techniques comes from the possibility to produce complex elements in a faster and easier way. Here, the component embeds the whole water circuit for the cooling of the TEMs surface and a small outside cave is used to seal the aluminum plate to the block. At the cold side, the heat sink is fed with water kept at 20 °C and a flow rate of 0.4 l/min by means of a chiller (Lauda EN252 thermostatic bath). Six aluminum layers are sealed to the ABS parts with transparent silicone adhesive sealant as an interface between the water fluid and the TEMs. This metallic layer promotes the homogenization of the surface temperature and an efficient heat transfer between the TEM surface and the cooling water. A photograph of a sector of the cold side heat exchanger (ABS heat sink and Al layer) to be coupled to the module is reported in Figure S3.

#### Assembled TEG

The whole TEG system assembled with an adjustable circumferential spring mechanism placed around the circular heat sink to apply uniform pressure on all TEMs. The pressure, calculated according to^25^ is 1 MPa on each thermoelectric module. Such a system allows the application of a constant pressure when the thermal expansion of the different elements of the thermal chain takes place, preventing to achieve a critical load to the TEMs. In addition, the heat sink components are designed to prevent any shear force to the TEMs converting the tangential clamping action into radial compression. To improve the thermal contact between the components of the thermal chain, a thermal paste has been used at the cold side of the TEMs, whereas thermal pads have been preferred at the hot side. The use of grease at the hot side, even if investigated, has been avoided due to the loss of performance in thermal cycling. The temperatures reached at the hot surface promote the evaporation of the fluid part of the paste leaving a cement poor in terms of thermal contact due to its reduction of performance in matching the surface evolution associated to the changes of temperature. A photograph of the entire experimental setup is reported in Figure S4.

### TEG system characterization

The characterization procedure is fully described in^26^. The TEG is investigated at different feeding rates leading to different ΔT. For each propane feeding rate, measurements are at the thermal equilibrium of the system: the evaluation of the steady state condition is based on the open circuit electrical output of the TEMs.

#### Temperature and voltage acquisition

Temperature measurements are performed with K and J type thermocouples at hot and cold sides for the TEMs, at the water inlet and outlet, and at the gas inlet and outlet. Thermocouples are embedded in the system in order to prevent possible effects on the thermal coupling between the surfaces of the thermal chain. Besides, to have a precise ∆T measurement, the open circuit voltage of all TEMs is converted to temperature differences using the standard characterization described in our previous work^26^. The overall system ∆T is obtained as the average of these values. Moreover, the hot/cold side temperatures are measured for two TEMs to further verify the measurement accuracy. The temperature at the bottom of the combustor is measured using a leaf thermocouple. The accuracy of calibrated K type and J type thermocouples is about ± 0.1 °C for the range between 0 and 300 °C and ± 2 °C for the range between 0 and 600 °C.

Following the detailed temperature characterization, the inlet and output power and, consequently, the efficiency of the TEG system is calculated as described in^26^.

The temperature and the voltage measurements are performed with an Agilent 349702A data logger and an Agilent 6060b electronic load. The data acquisition of temperature and electrical parameters is carried out with a LabVIEW control program to perform automatic measurement cycles.

#### Electric output

The main characterization approach is the rapid steady state^26^. Measurements are performed under constant ΔT condition: the fast load application (for about 20 ms) enables the electrical output regime keeping the thermal equilibrium state of the system unchanged. The resistive loads are sequenced from open to close circuit to carry out the measurements without considerable temperature changes. This method gives the maximum system performance for the TEG^27^.

A second characterization procedure used in this work is based on constant power input. In this case, the load application time is long enough to let all the thermal effects associated with Peltier and Joule effects occur. The performance results are lower, since this effect is due to changes in the effective ΔT at the TEMs. In this case, the best output can be achieved by increasing the fuel input, which means restoring the starting open circuit gradient. These analyses provide the effective output of the TEG for the design of an application based on the TEG under power supply operation mode.

#### Thermal output

The power components related to the inlet and outlet gases are also calculated. For the input power the lower heat value (LHV) of propane is used, while the power associated with the exhausts is calculated by measuring the temperatures at the different stages. As for hot temperatures, the gas combustion temperature value is considered. The temperature at the bottom surface of the TEG is measured in order to evaluate the calculation approximations: the system surface is isolated by refractory material minimizing the thermal losses from the lower part of the system.

Considering the geometry of each part, the convective coefficients for the circular heat sink, the TEMs and the combustor are set at 3, 15 and 10 W/m^2^K, respectively. For the emissivity coefficients of ABS material, stainless steel 316L and TEMs the values of 0.9^26^, 0.5^28^ and 0.6^29^ are considered, respectively. For both contributions the temperatures considered for the reference thermal gradient are the average temperature of the component measured during the experiment and the environmental temperature monitored during the sessions.

#### Chemical efficiency

Chemical efficiency is evaluated by measuring the gas species concentration at the exhaust of the combustor with Fourier Transform Infrared Spectrometry (FT-IR) as described in^30^. After passing through a water trap and a cut-off particulate heated filter, the exhaust gases are sent to the Thermo Scientific Nicolet 6700 FT-IR spectrometer equipped with a variable-path length heated gas-cell (Gemini Mars series 6.4 M, internal volume of 0.75 l). The sampling/transfer line and the gas-cell are kept at 393 K to prevent possible carbon-residuals condensation. The FT-IR spectra are collected with 0.5 cm^−1^ resolution and corrected for the background obtained with an N_2_ flow. The calibration procedure is performed before every measurement session with a known gas mixture composition in order to improve the data reliability. The analysis is performed at the maximum power production state where the load matches the TEMs internal resistance. At the same time, a continuous thermal and electrical data acquisition are carried out to detect the system performance variation and to measure maximum power production in the steady state condition.

## Results and discussion

**Figure 4:**
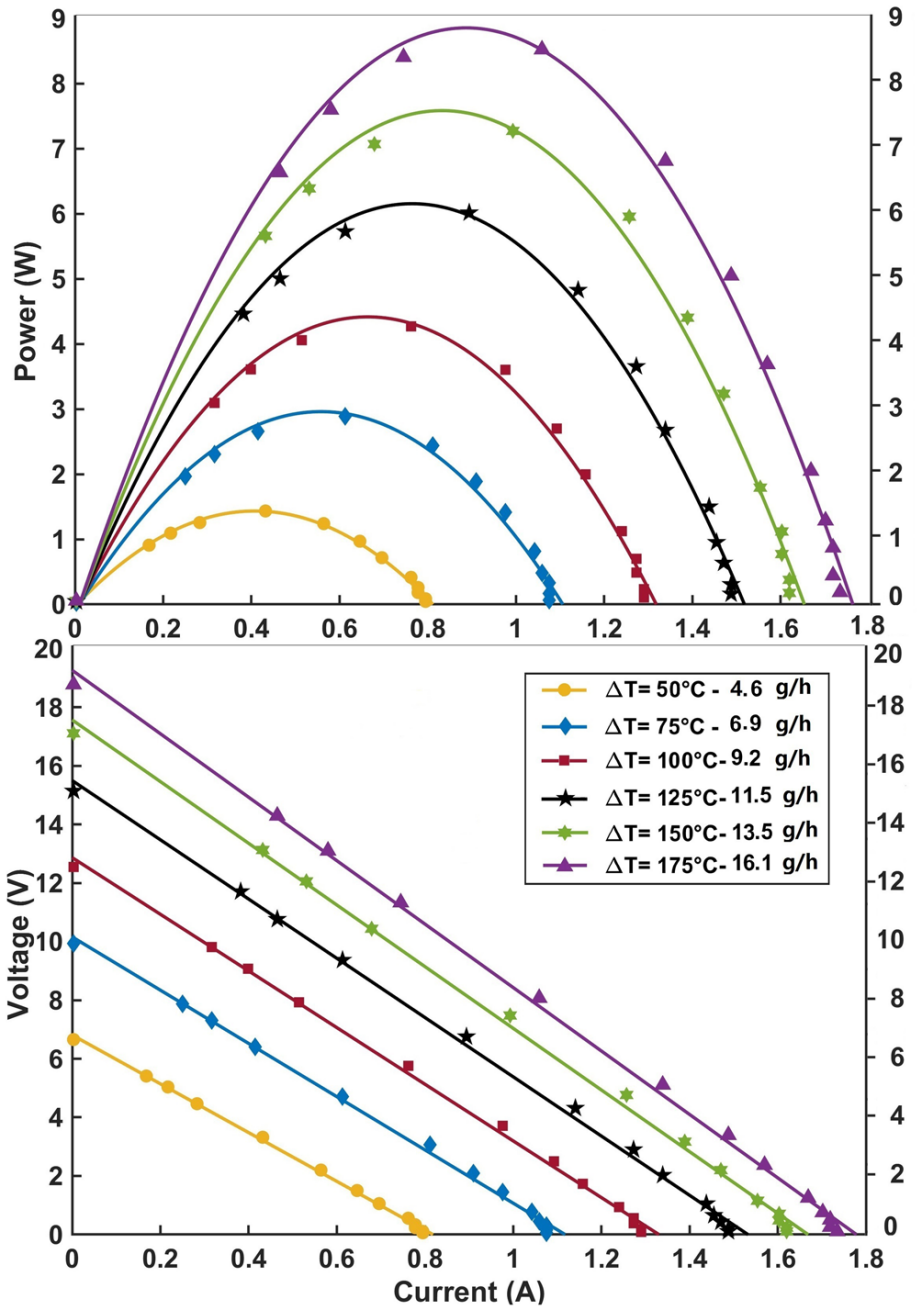
Electrical outputs characterizations of the TEG. The upper curves describe the electrical power produced versus current at different fuel flow rates. In the lower plot, the corresponding I-V characteristics of the TEG are shown. Data are collected from the six TEMs connected in series: no signal conditioning is applied in order to optimize the contribution from the different sides.

TEG electrical characterizations are shown in Fig. 4. Voltage-Current and Power-Current graphs are reported for different propane consumption rates at stoichiometric condition. The temperature differences ∆T reported in the graphs are obtained as an average of the temperature difference values of all six modules. Steady state condition is obtained in 10–15 min after combustion ignition and water cooling activation. Higher flow rates require more time to achieve the steady condition. Moreover, the lower the mass of the TEG components, the shorter the time to reach the steady condition. In particular, the combustor works as a buffer for heat during the electrical characterizations.

The results refer to the characterization with six modules in series. Considering the constant ∆T characterization of the TEG system, the maximum power produced with the 16.1 g/h propane consumption is 8.7 W (8.1 V, 1.1 A). At this mass flow rate, the hot side temperature is 220 °C. It is important to specify that no maximum point tracking system is applied and not even an optimization of the electrical output is attempted independently for the module electrical performance. This approach affects our results due to the differences in the applied ΔTs because of the differences in currents produced by each TEM. As a consequence, the approach here presented is the easiest method, even if not in an optimized configuration.

Keeping the system at the maximum load condition requires about 35 W of power, that is 2.6 g/h propane consumption at this flow rate, to compensate for the changes in heat resistance induced by Peltier, Joule, and Thomson effects.

Due to the series electrical connection of TEMs, the open circuit state output corresponds to the sum of the six module outputs. However, as discussed above the overall output, in the operating condition, results to be underestimated if compare to the sum of each module output. This can be explained by the fact that TEMs current inhomogeneity is responsible for some output drop^31^. Moreover, considering the measurement of each single module ∆T, a maximum difference of 25% has been obtained, which resulted in the same amount of output differences. The discrepancies in the TEG operation are mainly due to the combustion gas distribution and the pressure system inhomogeneity.

Being the total weight of the TEG system 350 g, the resulting power/weight production of the system is 24.8 W/kg. This ratio is significantly high and, to our knowledge, no other devices are described and proposed in the literature with such a high value. This result is primarily ascribed to the use of ABS polymer and the related low weight of the heat sink.

**Figure 5:**
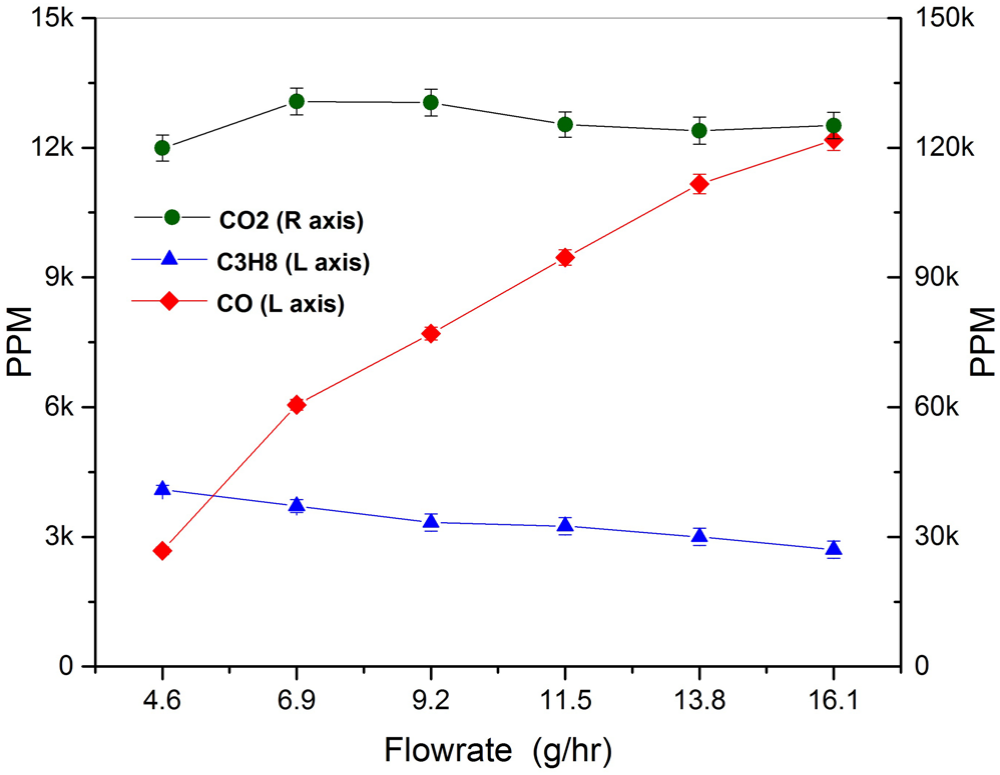
Carbon mono, di-oxides and residual unburnt propane concentrations versus the total flowrate. For each measurement the corresponding error bars are given.

The TEG chemical efficiency has been evaluated by measuring gas species concentration at the exhaust via FT-IR analysis. In Fig. 5 the concentration measurements of unburnt propane, carbon monoxide and carbon dioxide are reported as detected at the system outlet. The error bars are given as the standard deviations obtained by the FT-IR measurements. Considering these uncertainties, an error of 4% is obtained for the chemical efficiency evaluated at the different flow rates under analysis. Increasing the flow rate, the propane concentration constantly decreased up to about 30% from its initial value at 4.6 g/h propane consumption with 4093 PPM. By contrast, the carbon monoxide showed an increasing trend up to more than three times its initial value at 4.6 g/h propane consumption with 2680 PPM. The carbon dioxide presents a uniform trend with the flow rate, with a slight initial increase up to its maximum value of 130,696 PPM at 6.9 g/h and then a slight decrease to reach the value of 125,119 PPM at 16.1 g/h mass flow rate.

**Figure 6:**
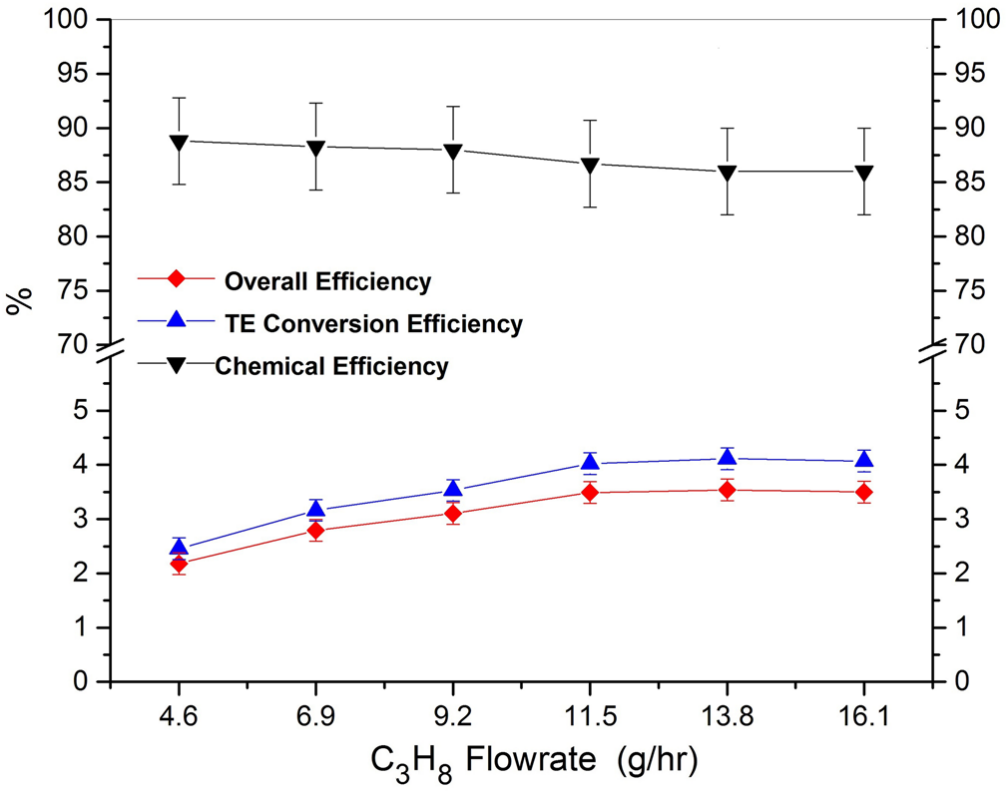
System conversion efficiency analyses. Chemical, thermoelectric (TE) and total efficiencies are reported for different fuel flowrates. The results are calculated from the heat flow measurements performed along the thermal chain of the generator.

The TEG system efficiencies are reported in Fig. 6, obtained as the ratio of the power input and the power produced by the system at the maximum load condition. Since the overall efficiency is the product of the chemical and thermoelectric conversion efficiencies, these values are also reported for each mass flow rate.

In the plots, error bars are also shown as calculated by considering gas concentration uncertainties evaluated in order to have the best and the worst case scenario. The chemical efficiency results to be 88.8% at 4.6 g/h and it remains almost constant for higher flow rates. A very slight decrease in the chemical efficiency can be attributed to the incomplete combustion of the gas mixture due to the lower gas residence time inside the combustion chamber. In fact, as it is shown in Fig. 6 the simultaneous increase of CO and decrease of CO_2_ is evidence of incomplete combustion.

The errors reported for the thermoelectric conversion efficiencies are related to the precision of the characterization instruments. The thermoelectric conversion efficiency ranged from 2.5 to 4.1% increasing the gas flow rates. The conversion efficiency of the TEMs is strictly dependent on their material Seebeck coefficient to the temperature and it increases for higher temperatures. The present thermoelectric efficiencies can be improved by means of a better coupling, and in fact an efficiency of up to 4.8% has been reported in the previous work^26^. Actually, the main difficulty in the system setup is obtaining a uniform pressure for all the TEMs by the circumferential spring. The overall efficiency of the system resulted to be between 2.2 and 3.5%. The maximum efficiency of 3.5% is the highest value reported in the literature for a TEG system based on catalytic combustion. This high overall efficiency is obtained essentially thanks to the novel design of the combustor proposed in the present work. In fact, although the size of the device is not substantially increased with respect to the device proposed in^26^, the new design strategy of the overall structure of the combustor and the new solution here proposed result to be effective to obtain a significant value of the system overall efficiency. Another issue of the present TEG is the indication of a correspondence between the maximum efficiency at the maximum power production. As it is also reported in the introduction and following the results presented in^21^, this result is very convenient from an economical point of view, being the present TEG system without a cost-free heat source.

Since the measurements are performed at the maximum power production, a lower chemical efficiency and consequently lower thermoelectric conversion and overall efficiency values can occur due to the unfavorable Peltier, Joule and Thomson effects, which become significant for high flow rates. At 16.1 g/h consumption, a temperature drops up to 40 °C has been observed after resistive load application at the TEMs hot side. The electrical output also decreased from 8.7 W (8.1 V, 1.1 A) to 7.3 W (7.3 V, 1 A), but this is a characteristic of all TEGs. As previously reported, an estimation of the power input needed to compensate for this effect has been performed obtaining a value of 18.7 g/h for the fuel consumption.

For a better characterization, the power balance is calculated for the present system. Based on energy conversion, the power input to output ratio has to be 1 for each fuel flowrate. Power input is calculated based on the LHV of propane. This value has to be scaled by the combustion efficiency obtained from FT-IR analyses. The output thermal power has been estimated from the contributions of radiation and convection and considering the power associated with the exhaust gases and the power removed by the water cooling system. For this purpose, the different mass flow rates used during the experiments and the temperatures measured in each operating condition are considered. For the evaluation of the residual thermal power at the exhausts, the gas mixture has been treated consisting only of air: this is a good approximation, being the air the main responsible for the thermal power removal from the internal chamber. In steady state condition, all the contributions associated with the components heat capacities can be neglected: these factors are involved in the inertia of the system but not in the heat transport phenomena. The data are reported in Table 2. The results show a relevant discrepancy between the input and the output powers, which scales down with the increasing flow rate.

**Table 2:** Power output and input collected at different fuel flow rates. The data refer to open circuit condition, then no electrical power is produced by the TEG and Peltier and Thomson effects are neglected. Moreover, the power loss in percentage is also reported for the fuel flow rates under analysis.

Flow rate, NL/min	Power, W					ΔP/P_in_ %
	Water	Exhausts	Convection	Radiation	Input	
0.5	11.0	1.5	2	0.9	27.4	44.0
1	28.3	3.0	3.5	1.8	52.6	30.4
1.5	37.7	4.6	5.5	3.1	78.4	34.8
2	50.2	6.1	6.5	4.4	104.2	35.0
2.5	62.8	7.7	8.5	6.6	128.4	33.1
3	80.1	9.1	10.5	8.0	152.8	29.4
3.5	94.2	10.7	12.5	10.7	178.3	28.7

This difference in the power output has to be searched in the superimposition of different issues related to the thermal chain of the system and to the heat flow management. Considering the components performances and the system design, the TEG electrical output was expected to achieve up to 12 W, while it reaches only a value of 9 W. This is partially due to the limited efficiency in transferring the whole heat produced by the catalytic combustion to the converting chain. The low speed of the hot gas associated to the flowrates reduces the efficiency of the heat collector at combustor side. Moreover, the chamber design, together with the material characteristics (limited thermal conductivity and the presence of surface porosity), promote the formation of a transversal temperature gradient affecting the performance of the modules. Such discrepancy can be pointed out by looking at the temperature data collected across the TEMs, reported in Table 3. The ΔT has been collected for each characterization in two ways. As a first analysis, two thermocouples have been positioned at the hot and cold surfaces in correspondence with two of the TEMs of the system. The two thermocouples are coupled with the borders of the combustor side face, for the hot side, and the Al plate, for the cold side. As a second approach, data are collected by the open circuit voltage of each TEM. The data underline a relevant difference between the temperatures applied to the TEMs and the effective ones, reaching a value of 35% for low flow rates. At low flowrates, the negative effects associated to the limited convective efficiency at the heat collector are larger as respect to the ones at higher flowrates, so the temperature data are out of the expected constant trend associated to the thermal resistances of the system, acting in the same way at every condition.

**Table 3:** Data collected for ΔT. The nominal data refers to the temperature collected using thermocouples placed at the hot and cold surfaces. The data for the modules are the converted values corresponding to the open circuit voltages of the single modules: these data are the average of the results obtained from the six TEMs.

Flow rate NL/min	ΔT_nominal_ (T_h_−T_c_) °C	ΔT_modules_ °C	ΔT_difference_ °C	ΔT_diff_/ΔT_nom_ %
0.5	16.5	26	9.5	37
1	40.7	55	14.3	26
1.5	66.0	80	14.0	18
2	87.5	105	17.5	17
2.5	105.8	130	24.2	19
3	122.2	149	26.8	18
3.5	144.8	174	29.2	17

The high thermal resistance observed has been explained considering two main critical aspects. The first, although the system is designed to transfer a radial pressure of 1 MPa to the six faces, a poor coupling of the pressure applied to the thermal chain could occur. Such mismatch in the coupling to the hot chamber could finally result in an inhomogeneous temperature difference for the different faces, as observed in the data collected on the six TEMs open circuit voltages. This is a technical issue associated to the latch used for the pressure application, unable to keep a circular shape when closed to the optimal shape. Another aspect to be considered related to the poor coupling, less relevant in terms of the effects but more difficult to overcome, is the surface finishing related to 3D growth technologies. In fact, the surface microstructure presents a strong roughness. In order to achieve a better finishing, the chamber's external faces have been polished to improve the thermal contact. Although such a procedure has been performed, a residual porosity of the material still remained, which results in a still significant contact resistance between modules and the heater, producing a relevant heat loss along the chain. This problem becomes critical as a consequence of the inhomogeneous pressure applied to the six sides: a proper pressure should allow overcoming such issue thanks to the use of interlayer of graphite to fill the gaps left by roughness and porosity between the modules and the heat collector.

Despite the discussed issues, there is still a power missing in the overall balance. To determine the possible ways of power losses, thermocouples have been placed in the insulating layers used to prevent heat exchange with external environment. The resulting analyses suggest that a relevant heat goes to warm up the basis of the device. In fact, the bottom surface of the combustor, actually insulated by a refractory shield reaches temperature up to 500 °C. The high heat capacity of the material used for the insulation promotes a large power absorption with a limited effect on average temperature of the shielding bulk. However, large local thermal gradient has been observed on the thermal shield supporting the hypothesis of its main role in power losses. In fact, such heat flow changes with time and temperature gradient between the combustor and the insulating shield, reaching a regime condition. Such effect would be also consistent with the major relevance at low flowrates, where the limited gas speed promotes a longer permanence of hot products of the combustion in the bottom region promoting the heat flow out of the system.

As a concluding remark, a short comment on the effective available electric power is necessary: all the analyses are performed without considering the power loss for the water pumping in the cooling circuit. The pumping system used for the experiments is integrated into the chiller and no specific details for its consumption are available. However, the requirements of the cooling system in terms of flow rates and volume capacity are compatible with common commercial pumps operating with electric power consumption in the range of 2 ÷ 5 W. As a consequence, the final net power produced by the TEG in the actual state can be evaluated in the range of 3.5 ÷ 6 W.

## Conclusion

A portable catalytic TEG based on a hexagon-shaped burner is developed and produced with AM manufacturing techniques. This work aims to be a starting point proposing a novel development approach to the design and production of thermoelectric generators for a large-scale use. Here a prototypal solution is presented operating in a 10 W electric power range, which can be of great interest for electronic devices power supplying or Li-ion battery charging. The AM tool results to be profitable in the production of customized components. In this work, two techniques have been used: SLBM and FDM for steel and ABS, respectively. AM allowed the shaping of the components producing light and complex structures capable of improved performances in the operating device. Moreover, such tool is very interesting and useful as a fast prototyping process for the development of a pre-industrial prototype. In particular, the main result of the present work is the high combustion efficiency reached, thanks to the key aspect of this work, related to the capability of coupling the inlet and outlet gasses, recovering the wasted heat, and lowering the catalytic combustion activation energy.

Another key point of this work is the choice to use an insulating material like ABS for the heat exchanger. The target of a light TEG has been achieved thanks to this strategy and was allowed by the possibility to produce an integrated water circuit into the printed component. This approach led to a compact and light device characterized by a high output power density.

Many considerations are taken into account to improve the system efficiency and performance. The TEG overall efficiency is evaluated to be between 2.2 and 3.5% depending on the gas mixture mass flow rates; the power produced ranges between 1.3–7.3 W with 4.6–16.1 g/h propane consumption, resulting in a max overall efficiency of 3.55%. These parameters correspond to long time regime operating conditions, here tested in order to evaluate the reliability of the system. In fact, the use of materials like ABS in high temperature ranges required a deeper investigation of the long lasting operating cycles. In the case of fast characterization, a maximum power output of 8.7 W is obtained with a power density close to 25 W/kg.

An additional consideration can be reported for the full combustion of the propane: it can deeply affect the overall performance of the device. In fact, despite an increase of the internal temperature is not desired, a more efficient combustion would allow a lower fuel consumption to achieve the same heat input. This represents a key aspect for the system promoting a longer operating time with the same hydrocarbon fuel feeding. In order to achieve this result, a further optimization of the system design has to be considered: in particular, a solution to improve the thermal exchange on the internal surface of the combustor has to be developed.

The thermoelectric device here presented represents a proof of concept of a new profitable strategy for portable energy solutions. The data collected allowed the identification of the further development still necessary to make the prototype a market ready product, with specific suggested solutions. New perspectives are actually under development for the optimization of the heat collection at the combustor wall and for the homogenization of the temperature profiles at the module surfaces. New design of the patterns both at hot and cold side should make the heat transfer more effective and promote a fuel saving to achieve the same output power. Such solutions will be based on technologies able to provide an improved density of heat transfer, in order to allow saving the size and the weight of the final device.

## Supplementary Information

Supplementary Figures.

## Data Availability

The datasets generated and/or analysed during the current study are not publicly available due to ongoing studies but are available from the corresponding author on reasonable request.
