# Supplementary material for: Additive fabrication and experimental validation of a lightweight thermoelectric generator
Source: Sci Rep. 2023 Jun 20;13:10042. doi: 10.1038/s41598-023-37222-w (PMC10281994; doi:10.1038/s41598-023-37222-w)
Supplement: Supplementary file 1 — Supplementary Figures. [file 41598_2023_37222_MOESM1_ESM.docx]

**SUPPLEMENTARY MATERIAL**


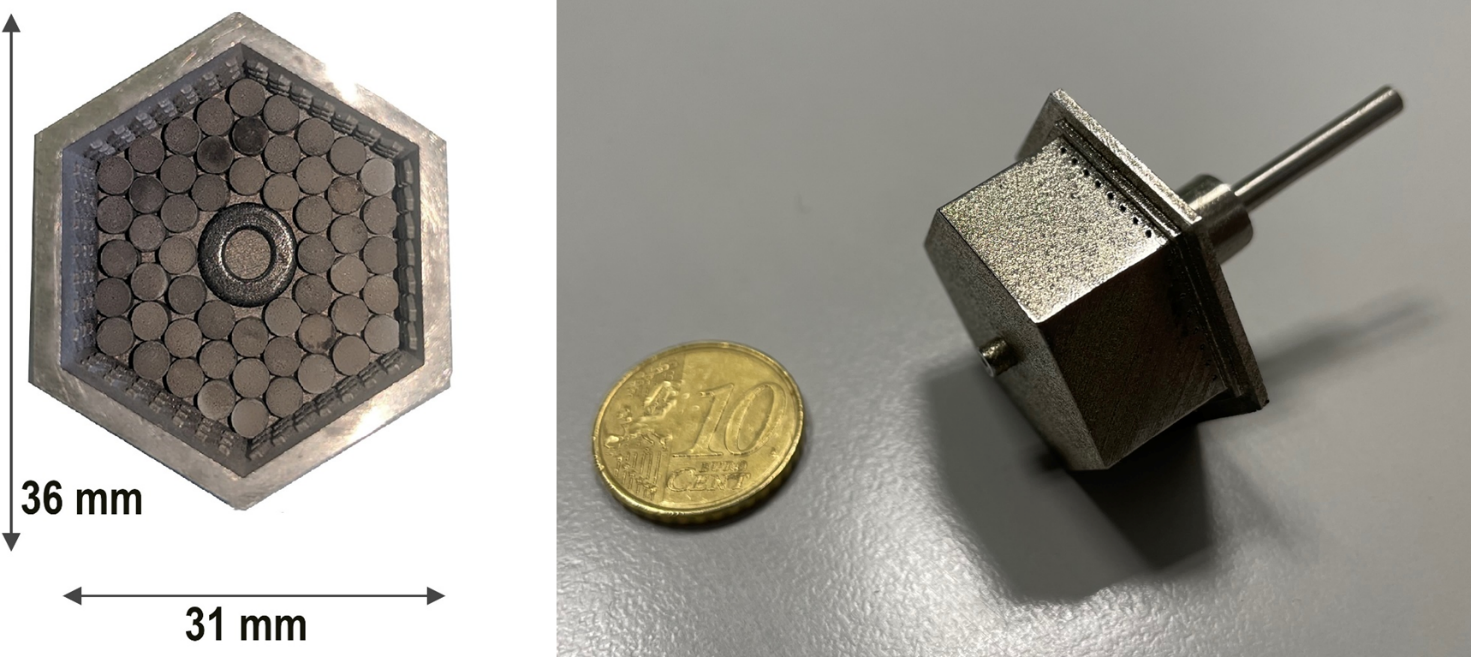


**Figure S1** Photographs of the inner side of the chamber with pellets on the base (left) and of the cap (right)

**
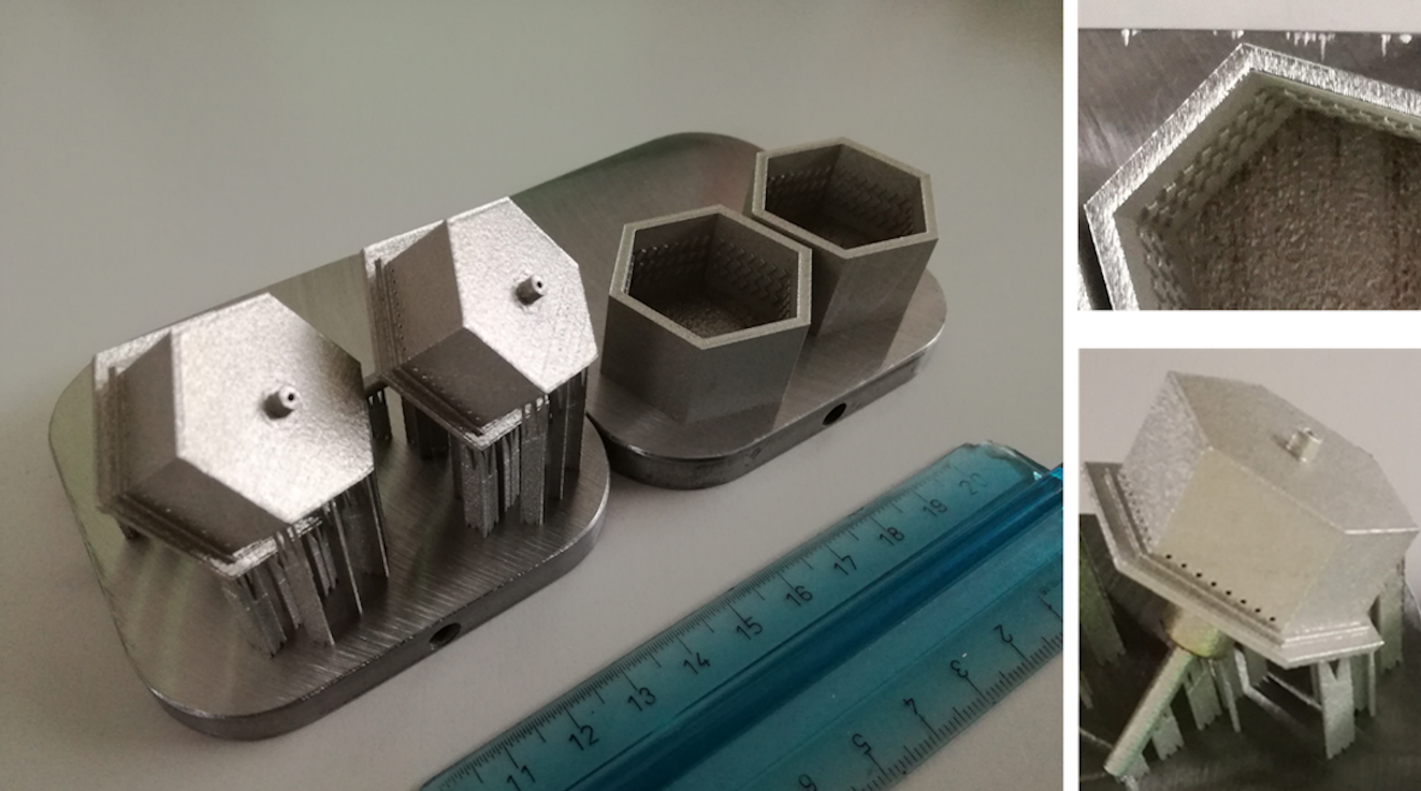
**

**Figure S2** Combustion chambers and caps on the building platform

**
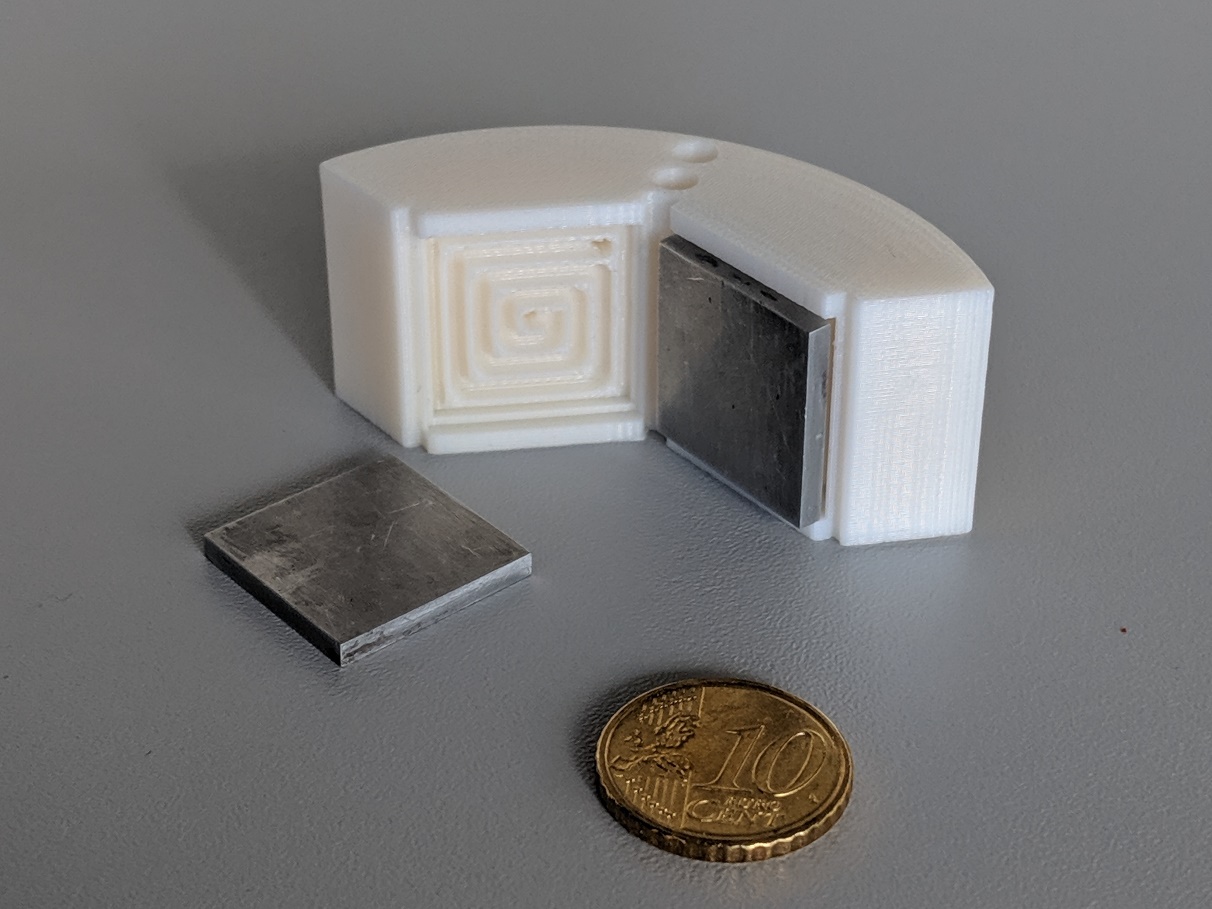
**

**Figure S3** Sector of the cold side heat exchanger (ABS heat sink and Al layer) to be coupled to the module

**
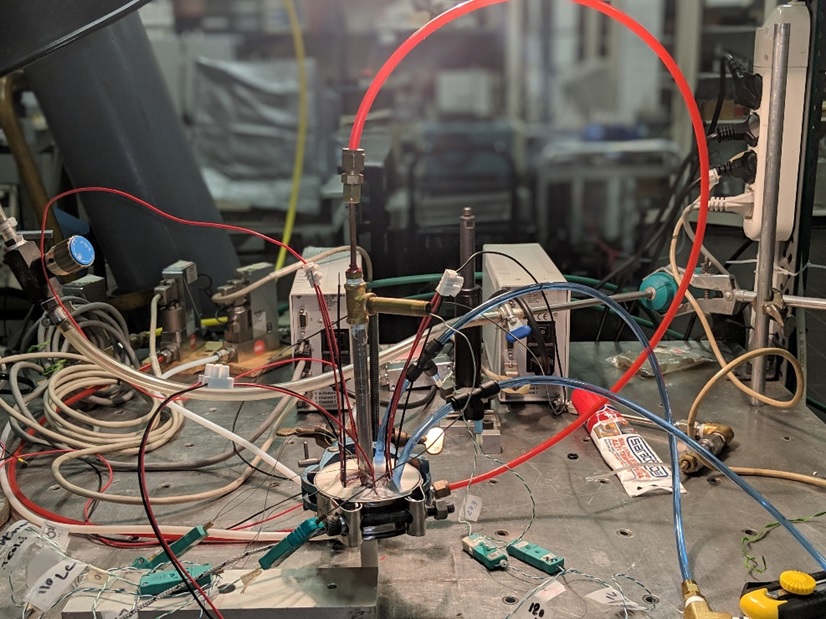
**

**Figure S4** Experimental setup: assembled TEG with the connections to the fuel feed system, the exhaust collector, the water cooling circuit and thermocouples.
